# Abstract of Proceedings of the Buffalo Medical Association

**Published:** 1868-07

**Authors:** T. M. Johnson

**Affiliations:** Sec’y.


ART. II.—*Abstract of Proceedings of the Buffalo Medical Association.*

TUESDAY EVENING, June 2d, 1868.

The meeting was called to order by the President. The report of the minutes of the last meeting was adopted. Members present—Drs. J. R. Lothrop, Rochester, Trowbridge, Hauenstein, Little, Phelps, Gay, Greene, White, Cronyn, Abbott, Samo, Jansen, C. F. A. Nichell, Smith, Wetmore, Potter, Kamerling and Johnson.

DR. C. C. F. GAY, as a delegate from this Association to the American Medical Association, made a brief report of the proceedings of the last meeting of the American Medical Association.

DR. HAUENSTEIN presented the following:—Mr. President, it appears that I have, unexpectedly, and entirely unsought by me, attained quite a degree of notoriety, the character of which, however, is anything but complimentary. I refer to the proceedings of a meeting of this Society, held in April, and which were published in the June number of the Buffalo *Medical and Surgical Journal*, which I received yesterday. On that occasion, it appears, my friend Dr. Gay, read a paper on the subject of Placenta Previa, in which he reported a number of cases, some of which had occurred in his own practice, and some in the practice of other physicians, to whose assistance he had been called. Among the cases reported are three, which occurred in my practice.

In the same paper, Dr. Gay stated the fact that in the brief period of eight months, six cases of placenta previa had occurred in my practice; this statement was rather too much for some of the members to believe, indeed, it so shocked their sense of professional propriety as to put them off their guard, and they permitted themselves to indulge in some most "*remarkably* remarkable" charges and insinuations.

Mr. President, I came here this evening to answer those charges, to repel those insinuations and to vindicate my honor. First, permit me to give a brief history of the cases as they happened in the order of time.

Case I.—The first of the six cases in question occurred on August 15th, 1867, and has been reported by Dr. Gay. The doctor will, however, please permit me to question the propriety of the use of the term "os rigid," which he uses. In order to enable the gentlemen present to judge for themselves as to the degree of rigidity of the os, at the time I entered it for the purpose of delivery, let me explain. I could readily enter the os with my hand, (the fingers held in the form of a cone,) to the heads of the metacarpal bones, when I found some resistance, but, keeping my hand *in situ* for a moment, it yielded and permitted it to pass comparatively easy. This was the degree of rigidity which it was necessary to overcome in order to get at the child in utero.

Case II.—November 2d, 1867, at 10 o'clock, P. M., was called to Mrs. S. H., on Batavia street, aged 39 years, in her tenth pregnancy at full term. Found her in a syncope, the pulse not perceptible; extremeties were cold and respiration feeble. On inquiry, I learned that three weeks ago she commenced flowing, and continued flowing with now and then two or three days of intermissions; the last six days had lost a large quantity of blood. Not knowing the danger of her condition, she would still, at times be up and about, attending to household affairs. Upon examination found os uteri dilated to size of a twenty-five cent piece, patient still flowing, and from appearance of bedding had already lost a large quantity of blood. Diagnosed placenta previa, inserted air-ball, (the first time I had ever made use of it in placenta previa,) administered stimulants, and sent for the assistance of Dr. Storck. No more loss of blood, yet no reaction took place, except a merely perceptible pulse; patient unconscious. Stimulants were freely given as also a dose of ergot. Feeble pains were occasionally observed, and at 1 o'clock, A. M., on the 3d of November, the airball being removed, the os was found dilated. Nature had wasted her strength and could not effect delivery unaided. Stimulants had accomplished nothing. To abandon her to her fate was death; we therefore concluded to deliver, as giving our patient the only chance of recovery. I introduced my hand and found placenta adherent to uterus all around; it was centrally implanted. After separating a portion from its attachment, to permit the passage in of my hand, the feet were brought down and the woman delivered. No apparent impression on the condition of the patient was noticeable; she had the same almost imperceptible pulse now as before. She lingered in this condition for two hours longer and died.

CASE III.—Mrs. S., on Walnut street, in the eighth month of her fifth pregnancy, was taken in labor with hemorrhage, on Friday morning, December 20th, 1867. A midwife was sent for who, finding herself inadequate to manage the existing complication, deserted the patient. Was called to see her for the first time on the evening of the next day, about 8 o'clock. Found patient had already lost considerable blood, none, however, during her pregnancy to commencing labor. Upon examination diagnosed placenta previa; os undilated. Inserted air-ball which completely controlled the hemorrhage. Remained with her all night, during which time she had feeble pains at long intervals. At about 8 o'clock in the morning pains increased, and blood began to flow rather freely. On removing the air-ball found os uteri dilated; introduced my hand for the purpose of delivering and found a segment of the placenta to her left side, and the feet of the child presenting; brought the feet down and delivered a still-born child; mother doing well.

CASE IV.—Mrs. B., on Madison street, near the end of the eighth month of her eighth pregnancy. Five weeks before confinement, after a copious loss of blood, came to my office to consult me. Three weeks afterwards had another attack of hemorrhage, and for the last week previous to her confinement, suffered from loss of blood every day or two. She had lost very much of blood before commencement of labor. February 8th, about noon, I was notified to attend her; found her in labor with feeble pains, attended with hemorrhage. On examination found the os uteri dilated, a segment of the placenta and the head of the child presenting. The hemorrhage being moderate, I gave her a dose of ergot and ruptured the membranes; to accomplish which, the membranes not being on a stretch during the slight pains she had, I introduced my hand into the vagina and ruptured them. The head soon engaged the inlet of the pelvis, after which she lost no more blood. Labor henceforth went on naturally and the child was born about 2 o'clock, P. M., and lived for five hours thereafter; mother doing well.

CASE V.—Occurred February 23d, as reported by Dr. Gay.

CASE VI.—The last case is also reported by Dr. Gay, and occurred on April 4th, 1868.

These are the cases to which Dr. Gay referred when he stated that six cases had happened in my practice in the short period of eight months.

Mr. President, in regard to the discussion which the treatment of my first case provoked, permit me to say a few words. In his remarks on the paper read by Dr. Gay, and in reference to the case where I delivered the woman, Dr. White—the first gentleman that spoke—said: "I am astonished, Mr. President, to hear the word 'forcibly' used, at this late day, to describe *any* of the manipulations practiced for the delivery of the parturient."

Mr. President, when a physician is called to assist the parturient, and from the nature of the case it becomes necess ry to make use of manipulations, more or less force will necessarily have to be used, and sometimes very considerable force must be used. Take, for instance, a case of transverse presentation, where, through ignorance, the midwife fails to detect the position of the child, and after the escape of the water and the lapse of many hours will yet administer one or several doses of ergot. To deliver such a woman requires force. To deliver a woman with forceps frequently requires the use of force for many hours in succession. Therefore, to deliver a woman forcibly need not necessarily be bad practice. But I agree entirely with the doctor when he modifies his assertion to cases when rigidity of the os uteri exists.

In the same paragraph the Doctor remarks: "The patient died, and as no *post mortem* is reported, it is impossible to determine the condition of the organs after this "forcible" resort to version of the child in utero." What is the simple meaning of this remark? Can it be construed into anything else but that I have made myself liable to a criminal prosecution? It is rather a strong insinuation. Dr. White is rather inclined to ridicule the practice of the use of the air-ball, and says that "it is not so good as soft old muslin, torn into small slips, and united by candle-wicking or tape, and then introduced through a cylindrical speculum." Mr. President, I consider the air-ball better than the tampon, which the gentleman is in the habit of using, based upon the fact that its introduction can be accomplished in a much shorter time, that no waste of time is necessary in its preparation, and that it completely fills the vagina. Whereas the tampon which he uses is open to the objection that when introduced through the speculum, each successive portion so introduced will be saturated with blood, and but an imperfect packing of the vagina can be accomplished. In future, however, I would accept of an improvement su gested by Dr. Greenhalgh, of London, which consists in surrounding it with a substance like spongiopiline, or, in the absence of which, I would use a piece of patent lint, so covering the ball as not to prevent its inflation.

"To expect the profession," said Dr. Miner, "to accept the declaration that one of these physicians has treated six cases of placenta praevia in eight months, is too much; it can hardly be credited; it must be looked upon as a mistake." The plain interpretation of this remark would sound somewhat harsh, I will therefore not attempt it. It is indeed strange that I should have had so many cases of placenta praevia in so short a time, I admit; but it is nevertheless a fact. I have no fault to find with the gentleman to be somewhat incredulous, but such is the language of expressing his incredulity that it conveys the insinuation of questionable veracity. The doctor's generosity, however, inclines him to be charitable, and he is forced to look upon it as a mistake. In all the six cases it was found necessary to introduce the hand into the vagina, and in the majority of the cases, into the womb; in all the six cases I performed whatever, manipulations were necessary, and I cannot comprehend the possibility of a mistake in the diagnosis, since I came in contact in each case with the placenta, and could feel it most distinctly—had it between my fingers. My practice in ordinary cases of midwifery is not large; I am called perhaps nine times in ten to cases where there is a midwife in attendance.

Dr. Gay said, it may perhaps be thought by some that there may have been an arrangement between Dr. Hauenstein and myself in the matter of this report, but there has been no conference or agreement in regard to what action should be taken in this matter.

Dr. White said that he did not intend to make any remarks, but would ask indulgence to say that he was glad to find that there is no difference of opinion between us. My remarks to which Dr. Hauenstein refers, were based upon what Dr. Gay said in his paper upon Placenta Previa. I supposed from the reading of his paper that forcible delivery was made while the os was rigid. The word forcible was used by Dr. Gay upon that occasion, and he admits here and now, that he used it. I use the terms *forcible* delivery and *artificial* delivery. I say that it is bad practice to undertake to forcibly deliver when the os is undilated and rigid. The use of the air ball is good practice, but not new at all. Accoucheurs differ in regard to the kind of tampon to use. I prefer the kitetail variety; others may prefer the air-ball. The number of cases of placenta previa reported by Dr. H. is large and very unusual, but they undoubtedly occurred. Dr. Hauenstein's veracity in the matter was not doubted—was not called in question, and the Doctor has this evening cleared up all the seeming differences of opinion in regard to the subject.

Scarlatina and rubeola were reported as prevailing.

Dr. J. R. Lothrop asked if it was the opinion of the members present that kidney affections oftener follow mild cases of scarlatina than severe cases. The prevailing opinion seemed to be that mild cases were most likely to be followed by these disorders.

Dr. J. S. Smith was elected to read an essay at the regular meeting of September next.

Adjourned. T. M. Johnson, Sec'y.

